# Identifying underlying individuality across running, walking, and handwriting patterns with conditional cycle–consistent generative adversarial networks

**DOI:** 10.3389/fbioe.2023.1204115

**Published:** 2023-08-04

**Authors:** Johannes Burdack, Sven Giesselbach, Marvin L. Simak, Mamadou L. Ndiaye, Christian Marquardt, Wolfgang I. Schöllhorn

**Affiliations:** ^1^ Department of Training and Movement Science, Institute of Sport Science, Johannes Gutenberg-University, Mainz, Germany; ^2^ Knowledge Discovery, Fraunhofer-Institute for Intelligent Analysis and Information Systems, Sankt Augustin, Germany; ^3^ Lamarr Institute for Machine Learning and Artificial Intelligence, Sankt Augustin, Germany; ^4^ Science&Motion GmbH, Munich, Germany

**Keywords:** cross-movement individuality, cross-signal individuality, CycleGAN, data augmentation, deep learning, generative adversarial network, movement pattern recognition, support vector machine

## Abstract

In recent years, the analysis of movement patterns has increasingly focused on the individuality of movements. After long speculations about weak individuality, strong individuality is now accepted, and the first situation–dependent fine structures within it are already identified. Methodologically, however, only signals of the same movements have been compared so far. The goal of this work is to detect cross-movement commonalities of individual walking, running, and handwriting patterns using data augmentation. A total of 17 healthy adults (35.8 ± 11.1 years, eight women and nine men) each performed 627.9 ± 129.0 walking strides, 962.9 ± 182.0 running strides, and 59.25 ± 1.8 handwritings. Using the conditional cycle-consistent generative adversarial network (CycleGAN), conditioned on the participant’s class, a pairwise transformation between the vertical ground reaction force during walking and running and the vertical pen pressure during handwriting was learned in the first step. In the second step, the original data of the respective movements were used to artificially generate the other movement data. In the third step, whether the artificially generated data could be correctly assigned to a person via classification using a support vector machine trained with original data of the movement was tested. The classification F1–score ranged from 46.8% for handwriting data generated from walking data to 98.9% for walking data generated from running data. Thus, cross–movement individual patterns could be identified. Therefore, the methodology presented in this study may help to enable cross–movement analysis and the artificial generation of larger amounts of data.

## 1 Introduction

After weak individuality had been used only sporadically for quite some time and, meanwhile, acquired the status of a buzzword, especially in connection with learning, training, and therapy, the investigation of strong individuality had increasingly been asked for (Schöllhorn, 1993; Bates, 1996). Often, a mixture of colloquial (weak) and science–oriented (strong) understanding can be observed, which is even more confusingly equated with “personalized” (Ginsburg and Willard, 2009; Ng et al., 2009; Chan and Ginsburg, 2011; Buford et al., 2013). Colloquially, weak individuality often serves as an excuse for a lack of statistically significant group differences (Davids et al., 1999; Button et al., 2000; Nuzzo, 2014) or for not finding commonalities across individuals (Schöner et al., 1992; Button et al., 2000; Hecksteden et al., 2015; Barth et al., 2019). In contrast, science on individuality is guided by the much stronger criteria of forensics, which must withstand legal proof for sentencing purposes. The two essential criteria are uniqueness and persistence (Jain et al., 2006; Kaye, 2010), for the proof of which, first, a larger amount of data is necessary, and, second, a different statistical method than the average oriented in social sciences is required. Both conditions explain why it is only with the more recent development of appropriate methods and techniques that the study of the strong individuality of selected forms of movement has increased almost inflationary. Originating from the visual perception of walking individuals (Johansson, 1973; Cutting and Kozlowski, 1977; Troje, 2002), followed by biomechanical analyses of gait movements (Schöllhorn et al., 1999; Windle et al., 1999; Schöllhorn et al., 2002) and sporadically single sports movements (Bauer and Schöllhorn, 1997; Schöllhorn and Bauer, 1998b), analyses of a wide variety of movements have become increasingly popular. Besides walking (Schöllhorn et al., 2002a; Begg et al., 2005; Begg and Kamruzzaman, 2005), the individuality of movements has also been shown in the field of sports in sprinting (Schöllhorn et al., 2001), running (Schöllhorn and Bauer, 1998a; Maurer et al., 2012; Hoerzer et al., 2015; Hoitz et al., 2021), javelin (Schöllhorn and Bauer, 1998b) and discus throwing (Bauer and Schöllhorn, 1997), and horseback riding (Schöllhorn et al., 2006), as well as in the field of music when playing the flute (Albrecht et al., 2014). Similar features could be shown for team behavior in volleyball (Westphal and Schöllhorn, 2001; Jäger and Schöllhorn, 2007), soccer (Grunz et al., 2012; Rein and Memmert, 2016), or basketball (Schmidt, 2012; Kempe et al., 2014). Typical parameters investigated are biomechanical data from video recordings (Kaur et al., 2023), force plates (Horst et al., 2016; Horst et al., 2017a; Horst et al., 2017b), pressure insoles (Schöllhorn et al., 2002b; Jacques et al., 2022), EMG (Jaitner et al., 2001; Aeles et al., 2021), and brain signals (Liao et al., 2014; Lee et al., 2022). Besides these, wearable sensors are becoming increasingly popular (Hammad and El-Sankary, 2020). Situated “perturbations” such as emotions (Janssen et al., 2008), fatigue (Burdack et al., 2020a), or time alone (Schöllhorn and Bauer, 1998b; Horst et al., 2016; Horst et al., 2017a; Horst et al., 2017b) were not able to move the patterns out of the strong individual space. Consequently, robust evidence for an important role of individuality is provided in short–term adaptive behavior. What all studies listed so far have in common is that they answer questions that could be carried out based on the comparison of a single movement technique. From this, the problem of individuality in longer–term learning, like in training or therapy, must be distinguished, especially with respect to the criterion of permanence. Repeating or learning the same movement by the same person never encounters comparable conditions again due to cognitive and body memory (Horst et al., 2020). To solve this problem, finding commonalities in learning different movements seems an appropriate approach but requires the identification of movement-independent individuality. In a first proposal, evidence for individual characteristics across three throwing techniques of the decathlon (final throwing phase of shot put, discus, and javelin) with similar kinematic structure was provided (Horst et al., 2020). The aim of this study is to identify individual commonalities of movement forms with different kinematic structures.

The problem with this is that the classification models cannot transfer among domains and thus only work on data from one domain. Consequently, it is not straightforward to train a classification model with walking data and test it on running data to identify any common underlying structures. However, new methods from the field of deep learning provide the potential to address this problem. Approaches from image generation offer solutions for analog problems. In image–to–image translation or style transfer, it is possible to learn a relationship between images from two domains, domain A (e.g., horses) and domain B (e.g., zebras), so that realistic images of domain B can then be generated from images of domain A. This has been done without losing the image content. Images of horses can become pictures of zebras, or images of a landscape can become pictures of the same landscape as it might look in summer or in winter.

In the area of data generation, Generative Adversarial Networks (GANs) (Goodfellow et al., 2014; Goodfellow et al., 2020) have proven to be extremely successful in generating new, previously unseen data that is somewhat similar to a given training data set. In order to solve “generative modeling” problems, the goal of a GAN is to learn the probability distribution of the data to be generated. Based on this probability distribution, the GAN then generates new data from this probability distribution. However, a major challenge with GANs is that they require a very large database of paired data to solve image-to-image translation (Zhu et al., 2017). This makes the GANs difficult to apply in the context of cross–movement studies for many problems. First, it is often not possible to generate the necessary large data sets, and second, due to the continuous change of movement patterns (Horst et al., 2019), finding matching pairs of movements between two movements can be problematic.

A further development of the GAN that circumvents the pairwise data problem that respectively offers a possible solution and can be successfully applied on relatively small data sets is the cycle–consistent GAN (CycleGAN) (Zhu et al., 2017). Here, images from domain A can be translated into domain B, but the basic content from domain A is preserved. A well–known example that makes use of this method is FaceApp (FaceApp Technology Limited, Cyprus). Given images of faces, the app allows for a transformation that makes someone’s face laugh, look older, look younger, or appear in the style of the opposite sex.

While the CycleGANs work quite well on images, it has not been applied to movement measurements. Therefore, in this work, we will use CycleGAN to identify common individual patterns across different movements. Specifically, we aim to find individual commonalities underlying the walking, running, and handwriting patterns of the same person.

With the CycleGAN, we generate artificial movement data of movement B from the original data of movement A. Specifically, we generate the other two movements from the walking, running, and handwriting movements (i.e., walking to running, walking to handwriting, running to walking, running to handwriting, handwriting to walking, and handwriting to running). The movements were chosen from the point of view that with walking and running, we have two related movements, and with handwriting, one very different from them. Based on former studies on individuality (Bauer and Schöllhorn, 1997; Schöllhorn et al., 2002a; Schöllhorn et al., 2002b; Schöllhorn et al., 2006; Janssen et al., 2008; Janssen et al., 2011; Schmidt, 2012; Albrecht et al., 2014; Horst et al., 2016; Horst et al., 2017a; Horst et al., 2017b; Horst et al., 2019; Burdack et al., 2020a), we assume that individuals can be distinguished by their walking, running, and handwriting patterns. From this, we derive the following research questions: Can CycleGANs artificially generate pairwise data between walking, running, and handwriting movements, and can this artificial data be assigned to the correct individuals?

## 2 Materials and methods

### 2.1 Participants and ethics statement

The study participants were 17 athletically active, healthy adults (eight women and nine men; 1 left–handed and 16 right–handed) who regularly handwrote and ran for health reasons (the group characteristics are shown in Table 1). Before participating in the study, the participants signed informed consent forms. The study was conducted according to the guidelines of the Declaration of Helsinki and approved by the Ethics Committee of the Johannes Gutenberg–University Mainz (2022/05; 5/23/2022). Each participant visited the biomechanics laboratory once, where all measurements took place.

**TABLE 1:** Participant characteristics.

	M	SD
Age (years)	35.8	11.1
Height (cm)	172.1	6.2
Body mass (kg)	68.0	9.3
Preferred walking speed (km/h)	4.2	0.5
Preferred running speed (km/h)	8.4	1.4

Data are presented as mean (M) and standard deviation (SD); preferred walking and running speed was determined while walking/running on the treadmill.

### 2.2 Experimental protocol

At the beginning of the study, the preferred walking speed (PWS) and preferred running speed (PRS) on the treadmill were determined for each participant (Dal et al., 2010). The PWS is a speed that the participants prefer in their leisure time, for example, when going for a walk, and the PRS is a speed at which they “feel comfortable” and “can keep going for a very long time”. At the same time, the determination of PWS and PRS also served as a habituation to the treadmill. This was followed by familiarization with writing on the digitizing tablet with a pressure–sensitive pen, with everyone writing the sentence that was also written in the data collection (see below) five times.

**FIGURE 1:**
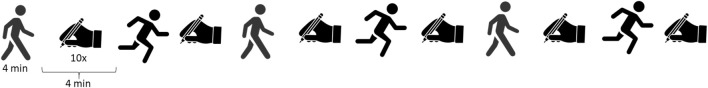
Experimental sequence with the chronological sequence of the three walking, three running, and six handwriting conditions. The walking and running conditions were always alternated with handwriting. In addition, walking and running were always alternated for stress control reasons. The starting condition (walking or running) was randomized between participants.

As presented in Figure 1, the participants performed six sets of 4 min of running or walking. Each was followed by a 4–minute break during which they performed 10 handwriting trials. To achieve greater variation within participants (for more robust training of the Deep Learning models, we increased the variance), we varied the speed in each of the three walks and runs slightly from slow: 85% PWS/PRS, to normal: 100% PWS/PRS, and fast: 115% PWS/PRS. To avoid sequence effects, we randomly shuffled the order of the walking and running conditions across all participants, with the only restriction being that walking and running must always alternate due to load control. Between each walk and run, the phrase “Wellen folgen den Bewegungen” [English: “waves follow the movements”] was handwritten 10 times. The sentence was chosen because it was as neutral and as contentless as possible in terms of meaning. Again, to provoke greater variation in the data, a new instruction was given for each handwriting set, which was implemented at the discretion of the participants. The instructions included writing “normal” or “bigger”, “smaller”, “faster”, “slower”, and “more beautiful” as usual. Again, we randomized the order of each instruction.

### 2.3 Data acquisition

The movements investigated in this study are walking, running, and handwriting. Walking and running were performed on a treadmill (cos12148, h/p/cosmos, Leipzig, Germany) and recorded with pressure soles (pedar, novel, Munich, Germany) at a frequency of 100 Hz. The handwriting was performed using a pressure-sensitive pen (Wacom Pro Pen 3D, Wacom, Düsseldorf, Germany) with 4096 pressure levels on a digitizing tablet on which a paper was adjusted (Wacom Intuos Pro Paper Edition L, Wacom, Düsseldorf, Germany) with a recording frequency of 200 Hz and recorded with the software CSWin (CSWin 2016; MedCom Verlag, Munich, Germany).

### 2.4 Data processing

For the locomotion tasks, the vertical ground reaction force (GRF) was calculated from the pressure data using Pedar Mobile Expert software (version 8.2). The stance phase from heel strike to toe–off of the left and right foot was determined using a vertical GRF threshold of 50N. Each ground contact with one foot was time-normalized to 128 values. Burdack et al. (2020b) showed that the exact vector length plays a minor role as long as the curve shape is preserved. In addition, the data were normalized by body weight and scaled to the range [0, 1] (Chau, 2001a; Chau, 2001b; Hsu et al., 2003). After scaling, the step pairs from the left and right ground contact were combined into one vector of 256 values (128 data points left foot + 128 data points right foot). Each vector begins with a left-ground contact and ends with the corresponding right one. If the data of ground contact was incorrect during recording, this and the corresponding ground contact of the other foot were deleted from the recording.

For handwriting, only the vertical pen pressure data where the pressure on the pen was greater than zero was considered. Furthermore, we considered only the first letter W for the handwriting analysis. If the W was not written in one piece, the test was discarded. In the case that the ‘e’ was written from the W without settling, the point with the least pressure between the W and the ‘e’ determined the end of the W. In addition, the handwriting data were filtered with a 1st–order Savitzky–Golay filter with a window size of 13 (Savitzky and Golay, 1964), which smoothens on the least squares method while maintaining the shape and height of the waveform peaks (Schafer, 2011). Data were also time–normalized to 256 data points to have the same length as the GRF data, z–standardized, and scaled to the range [0, 1]. The reduction of the entire sentence to the letter W had several reasons. First, preliminary measurements showed that compressing the signal of the entire sentence to 256 data points meant that the handwriting could no longer be generated sufficiently well. Derived from this, we wanted to obtain a signal that was similarly complicated and on a similar time scale in execution as that of the locomotion movements.

### 2.5 Data analysis

#### 2.5.1 Data analysis procedure

**FIGURE 2:**
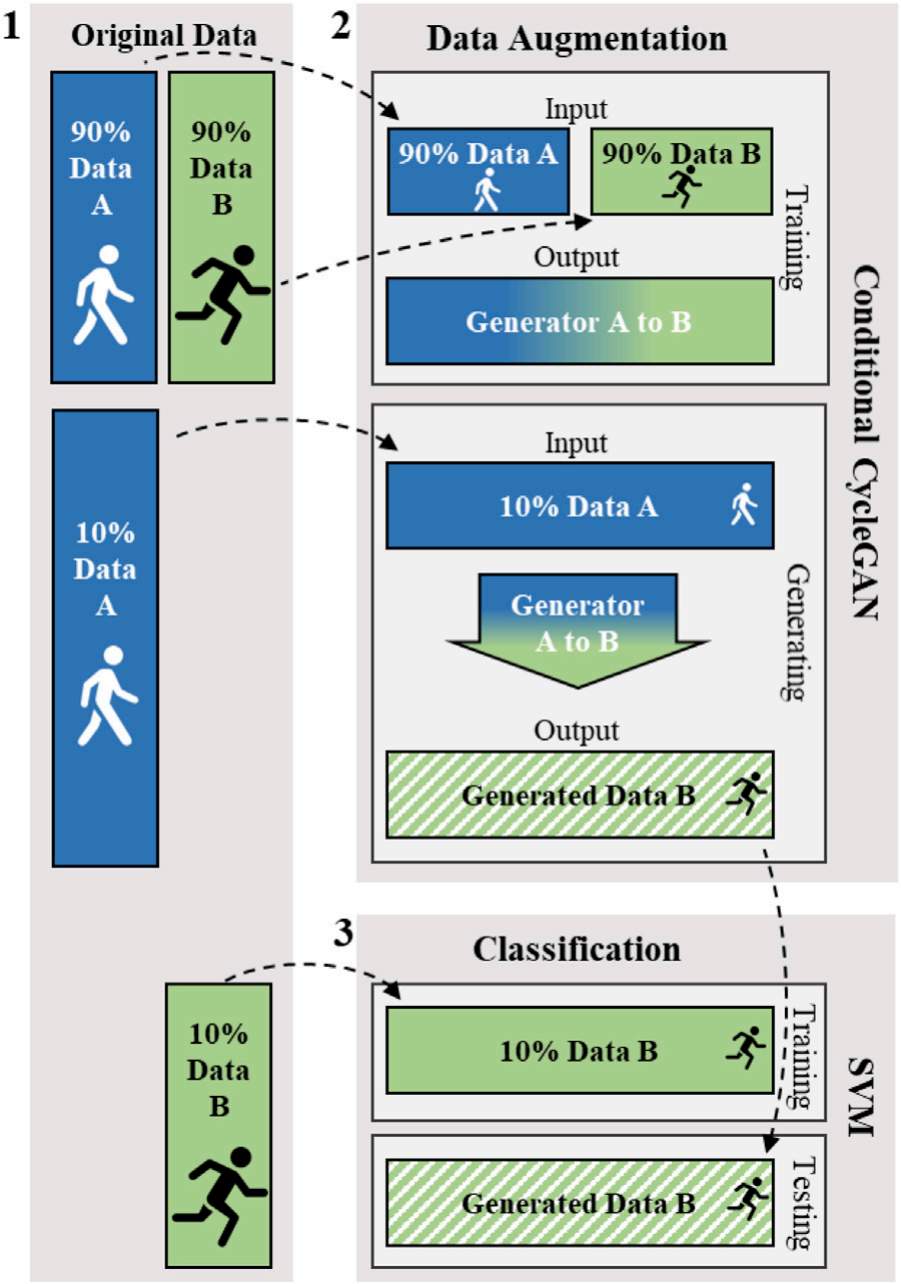
Description of the procedure from data generation to classification. For the sake of clarity, only the way of generating new data from data A is described in the figure. However, the way of generation from data B is analogous. For illustration purposes, data A corresponds to walking and data B to running. (1) Original Data: In each case, the original data was split 90:10. (2) The outlined process of the conditional CycleGAN: 90% of the data of A and B were used for training the conditional CycleGAN. The trained generators were then used to generate new data B from the remaining 10% of data A accordingly. (3) The outlined procedure of classification using a Support Vector Machine (SVM): the SVM was trained with the 10% of data B that was not used for training the conditional CycleGAN. In each case, the SVM was tested with the newly generated data B.

Figure 2 shows a schematic example of the data analysis flow for a portion of the data (Data A). It is important to emphasize that the conditional CycleGAN training data is separate from the generation data and from the SVM training data. While the conditional CycleGAN training for data A and B occurs simultaneously, the paths of data A and B are strictly separated from the time of data generation. The details of data generation and classification are described below.

#### 2.5.2 Data generating–conditional CycleGAN

With the CycleGAN (Zhu et al., 2017), we want to translate the movements of walking, running, and handwriting, keeping individual features in each case. Again, an unpaired approach is particularly appropriate because we want to translate data from one movement domain (e.g., walking) into data from another movement domain (e.g., running) without knowing a possible mapping of the different movements of each domain to each other.

In doing so, our approach deviates somewhat from the original CycleGAN formulation. Since we want to preserve the individual component via the movement data transfer as well, we have added a class condition to the conventional CycleGAN in the following.

##### 2.5.2.1 Formulation of the conditional CycleGAN

The goal of the conditional CycleGAN, conditioned on the participant’s class label, is to learn mapping functions between two domains X and Y under the condition of the classes Z given the training samples ${\left\{x_{i}\right\}}_{i=1}^{N}$ where $x_{i}\in X$ and ${\left\{y_{j}\right\}}_{j=1}^{M}$ where $y_{j}\in Y$ , and the class embedding ${\left\{z_{k}\right\}}_{k=1}^{P}$ where $z_{k}\in Z$ . For simplicity, the indices i, j, and k are omitted in the following. The data distribution is denoted as ${x\;∼\;p}_{data}\left(x\right)$ and ${y\;∼\;p}_{data}\left(y\right)$ , for the input of the original data, ${z\;∼\;p}_{class}\left(z\right)$ for the class embedding, and ${x,z\;∼\;p}_{data}\left(x,z\right)$ and ${y,z\;∼\;p}_{data}\left(y,z\right)$ for the data under the class condition. The conditional CycleGAN includes two mappings $G:\left(X,Z\right)\to Y$ and $F:\left(Y,Z\right)\to X$ . Furthermore, there are two adversarial discriminators $D_{X}$ and $D_{Y}$ , where the aim of $D_{X}$ is to distinguish between data $\left\{x\right\}$ and translated data $\left\{F\left(y,z\right)\right\}$ and correspondingly for $D_{Y}$ to distinguish between $\left\{y\right\}$ and $\left\{G\left(x,z\right)\right\}$ . In the following, the terms adversarial loss (Goodfellow et al., 2014; Goodfellow et al., 2020), cycle–consistency loss (Zhu et al., 2017), and identity–mapping loss (Taigman et al., 2017), which are elementary for the conditional CycleGAN, are described and finally summarized in the objective function.

Adversarial Loss (Goodfellow et al., 2014; Goodfellow et al., 2020): To both functions, adversarial losses are applied. For the mapping function $G:\left(X,Z\right)\to Y$ and its discriminator $D_{Y}$ , the adversarial loss is:

$$L_{GAN}\left(G,D_{Y},X,Y,Z\right)=E_{y,z∼p_{data}\left(y,z\right)}\left[\log D_{Y}\left(y,z\right)\right]+E_{x,z∼p_{data}\left(x,z\right),z∼p_{class}\left(z\right)}\left[\log\left(1-D_{Y}\left(G\left(x,z\right),z\right)\right)\right]$$

where G aims to generate data $G\left(x,z\right)$ that look similar to data from domain Y, while $D_{Y}$ tries to distinguish them from the real samples y . G tries to minimize this goal against an adversary D , which in turn tries to maximize it: $\min_{G}\max_{D_{Y}}L_{GAN}\left(G,D_{Y},X,Y,Z\right)$ . The adversarial loss for the mapping function $F:\left(Y,Z\right)\to X$ and its discriminator $D_{X}$ is formulated accordingly.

Cycle–Consistency Loss (Zhu et al., 2017): In addition, to reduce the space of possible mapping functions G and F, the mapping functions should be cycle–consistent. For each data x from domain X , the data translation cycle should be able to return x to the original data: $x\to G\left(x,z\right)\to F\left(G\left(x,z\right),z\right)\approx x$ , which is called *forward cycle consistency*. The *backwards cycle consistency* applies accordingly to y from domain Y . To achieve cycle consistency, the following cycle–consistency loss is expressed:

$$L_{CYC}\left(G,F\right)=E_{x,z∼p_{data}\left(x,z\right),z∼p_{data}\left(z\right),x∼p_{data}\left(x\right)}\left[∥F\left(G\left(x,z\right),z\right)-x{∥}_{1}\right]+E_{y,z∼p_{data}\left(y,z\right),z∼p_{data}\left(z\right),y∼p_{data}\left(y\right)}\left[∥G\left(F\left(y,z\right),z\right)-y{∥}_{1}\right]$$

Identity–mapping Loss (Taigman et al., 2017): To promote the successful reproduction of the input, an identity-mapping loss is formulated:

$$L_{ID}\left(G,F\right)=E_{y,z∼p_{data}\left(y,z\right),y∼p_{data}\left(y\right)}\left[∥G\left(y,z\right)-y{∥}_{1}\right]+E_{x,z∼p_{data}\left(x,z\right),x∼p_{data}\left(x\right)}\left[∥F\left(x,z\right)-x{∥}_{1}\right]$$

Full Objective: To summarize, the complete objective is:

$$L_{CYC}\left(G,F,D_{X},D_{Y}\right)=L_{GAN}\left(G,D_{Y},X,Y\right)+L_{GAN}\left(F,D_{X},Y,X\right)+{\lambda_{CYC}L}_{CYC}\left(G,F\right)+{\lambda_{ID}L}_{ID}\left(G,F\right)$$

where $\lambda_{CYC}$ and $\lambda_{ID}$ control the relative importance of the two objectives, respectively.

The goal is to solve the following equation:

$$G^{*},F^{*}=\arg\min_{G,F}\max_{D_{X},D_{Y}}L\left(G,F,D_{X},D_{Y}\right)$$

##### 2.5.2.2 Architecture and training details

The basic architecture and training details are based on the CycleGAN architecture of Zhu et al. (Zhu et al., 2017) and its implementation on GitHub (https://github.com/junyanz/CycleGAN). Thereby, the generator architecture is in turn based on the GAN architecture of Johnson et al. (Johnson et al., 2016), and the discriminator architecture is based on PatchGANs (Li and Wand, 2016; Isola et al., 2017; Ledig et al., 2017). We implemented the class conditioning according to the conditional GAN model of Isola and colleagues (Isola et al., 2017). To implement the code, we used Tensorflow 2.9.2 (Abadi et al., 2015). In the following, we point out all differences in specifications from the originally proposed constructs and training parameters.

**FIGURE 3:**
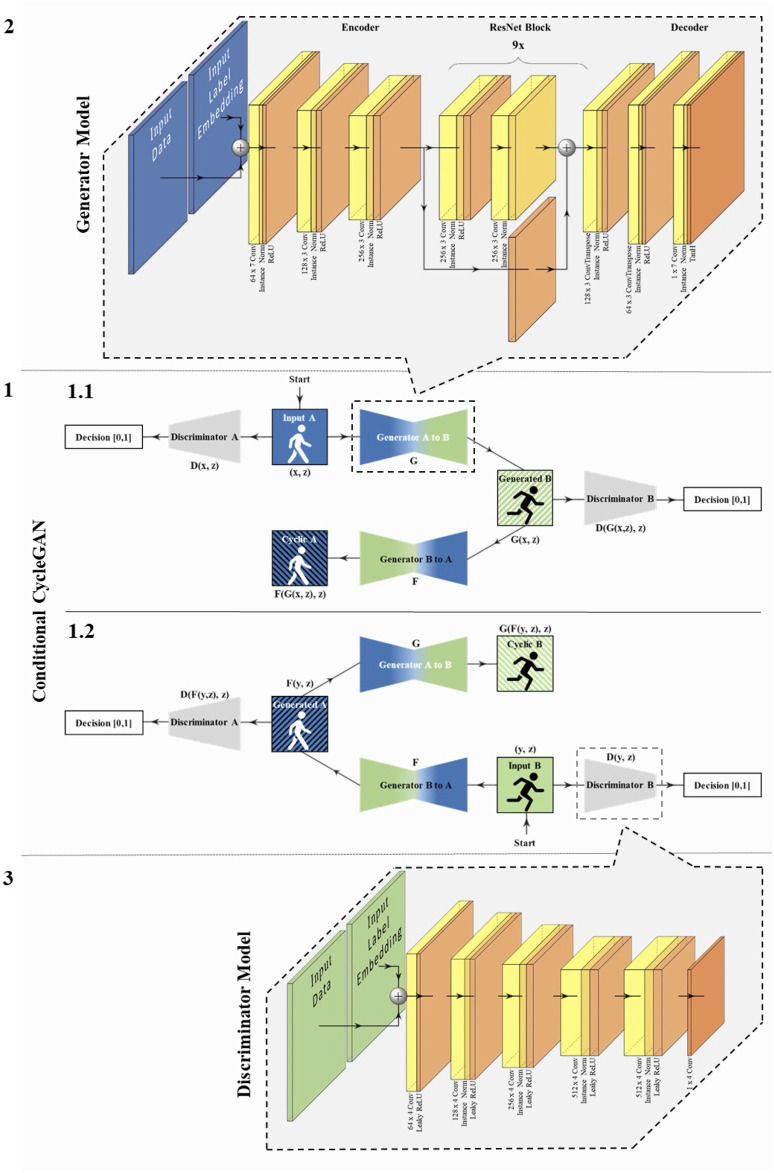
Schematic structure of the conditional CycleGAN, the generator, and the discriminator model. (1) The conditional CycleGAN with walking exemplary for Data A and running for Data B: In 1.1, the path is shown starting from Data A, and in 1.2 starting from Data B. It is important to emphasize that the identically named generators and discriminators are the same in each case and are trained from both directions. Furthermore, the basic generator and discriminator models are the same. (2) Generator model: presented are the layers including filter and kernel size (e.g., 64 × 7 Conv = convolutional Layer with 64 filters and a kernel size of seven). (3) PatchGAN discriminator model: presented are the layers including filter and kernel size.

The specific layers used for the generator and discriminator models, including their filter and kernel sizes, are shown in Figure 3. We fitted all layers to a one–dimensional input. Moreover, for the convolutional layers shown, we initialized the model weights with a random Gaussian with a mean of 0.00 and a standard deviation of 0.02. In addition, we used the same padding.

Other specific settings not shown in Figure 3 for the discriminator and generator are noted below. For the discriminator, we used 70 × 1 PatchGANs according to Isola et al. (2017). The convolutional layers had a stride of two for all layers except the output layer, where the stride is one. In addition, the slope of the leaky ReLU layers was set to *α* = 0.2. The generator model according to Johnson et al. (2016) consists of an encoder, nine consecutive residual networks (ResNet) for transformation, and a decoder. The stride of the first and last convolutional layers and ResNet convolutions is one, while it is two in every other convolutional layer. For all parameters of the discriminator and generator not mentioned, we used the default values of Tensorflow (version 2.9.2).

Furthermore, 200 epochs were trained with a batch size of 64. The discriminator used the Adam solver with a constant learning rate of 0.0002. While for the generator, we adjusted the learning rates according to the pair of data. For the conditional CycleGAN between walking and running data, as suggested in the study by Zhu et al. (2017), the learning rate was set to 0.0002 for the first 100 epochs and then linearly decreased to 0 for the next 100 epochs. For the conditional CycleGAN between walking and handwriting data or running and handwriting data, in the first 50 epochs, the learning rate was 0.0128 (= 0.0002 * 64 (batch size)); in the next 50 epochs, it was 0.0016 (0.0002 * 8 (square root of the batch size)); and in the last 100 epochs, it was linearly decreasing to 0. In addition, λ_CYC_ was set to 10 and λ_ID_ to 5 in Equation 4. The trained generator with the lowest loss value over each of the 200 epochs was selected for data generation.

##### 2.5.2.3 Data classification

Since there are no theoretical or practical empirical values for the time series-based method used in this study in terms of the minimum amount of data, we roughly followed the sizes of the image datasets used in the CycleGAN article (Zhu et al., 2017). We collected 10,661 walking strides (627.9 ± 129.0 per person), 16,358 running strides (962.9 ± 182.0 per person), and 1,067 handwritings (59.25 ± 1.8 per person). Each vector of walking or running stride and handwriting included 256 data points (walking/running: 256 data points = 128 data points of left food contact + 128 data points of the right food contact).

Participant classifications were based on support vector machine (SVM) (Cortes and Vapnik, 1995; Boser et al., 1996; Müller et al., 2001; Scholkopf and Smola, 2002) with an extensive hyperparameter search in terms of kernel (linear, radial basis function, sigmoid, and polynomial) and cost parameter (C = 2^−5^, 2^−4.75^,. . . , 2^15^).

We examined the performance to discriminate walking, running, and handwriting patterns between participants using a multi-class classification with 17 classes, where each participant represented one class. As shown in Table 2 for the original data (and in more detail for the generated data in the Supplementary Table S1), the amount of training and testing data used for the classifications varied in size due to the different data sets. In addition, to relate the results of the participant classification based on the original data, we exactly matched the training and test splits of the walking and running classification to those of the writing classification.

**TABLE 2:** Description of the data of the baseline participant classification by means of SVM based on the original walking, running, and handwriting data.

Original data	SVM	
	Number of training data	Number of test data
Walking	9,594 (565.1 ± 110.7) [905 (53.3 ± 1.5)]	1,067 (62.9 ± 12.3) [101 (5.9 ± 0.3)]
Running	14,722 (866.6 ± 163.9) [905 (53.3 ± 1.5)]	1,636 (96.3 ± 18.1) [101 (5.9 ± 0.3)]
Writing	905 (53.3 ± 1.5)	101 (5.9 ± 0.3)

An SVM with five–fold cross–validation was applied. The table shows the total number of trials for the training and test data from all 17 participants. The mean amount and standard deviations of the trials of each participant are shown in round brackets. In square brackets, the number of trials of the walking and running data adjusted for the number of trials of the writing data is also shown.

To evaluate the results of the multi-class classifications, the performance indicators accuracy, F_1_-score, precision, and recall were calculated after five-fold cross-validation. The number of true positives (TP), true negatives (TN), false positives (FP), and false negatives (FN) define these metrics:

$$Accuracy=\frac{TP+TN}{TP+TN+FP+FN}$$

$$Precision=\frac{TP}{TP+FP}$$

$$Recall=\frac{TP}{TP+FN}$$

$$F_{1}-score=2*\frac{Precision*Recall}{Precision+Recall}$$

The baseline reference is the zero-rule baseline (ZRB), which results from the theoretical accuracy when the classifier always predicts the most frequented class of the training set. Since our dataset is unbalanced, the ZRB is calculated by dividing the number of training trials of the most frequent class by the number of all training trials in the corresponding classification task:

$$ZRB=\frac{Training\;Trials\;Most\;Frequent\;Class}{All\;Training\;Trials}$$

For a detailed overview of the calculated ZRB for each classification task, see Supplementary Table S2.

The classification was performed within Python version 3.9.12 (Python Software Foundation, Wilmington, DE, United States) using the scikit–learn toolbox version 1.1.3 (Pedregosa et al., 2011).

## 3 Results

### 3.1 Participant classification on original data

The basic assumption of this study is the existence of the distinguishability of the individual movement patterns between the persons. To test this assumption, an SVM was used to classify individuals based on walking, running, or handwriting data. As presented in Table 3, it is possible to distinguish the participants from each other in both the walking and running data, and the handwriting data with more than 98.0% classification F_1_–score each.

**TABLE 3:** Classification scores of person recognition using SVM based on original data.

Classification problem	Classification score [%]					Number of trials	
	Acc	F_1_	Prec	Rec	ZRB	Train	Test
Walking	99.7 [98.0]	99.7 [98.0]	99.7 [98.4]	99.7 [98.0]	7.5 [6.0]	9,594 [905]	1,067 [101]
Running	99.6 [99.0]	99.6 [99.0]	99.6 [99.1]	99.6 [99.0]	6.7 [6.0]	14,722 [905]	1,636 [101]
Writing	99.0	99.0	99.2	99.0	6.0	905	101

In squared parentheses are the results of the original walking and running classification, where the number of trials was exactly matched to that of the writing classification. ZRB, Zero–Rule Baseline; Acc, Accuracy; F_1_, F_1_–Score; Prec, Precision; and Rec, Recall.

### 3.2 Qualitative analysis of generated data

**FIGURE 4:**
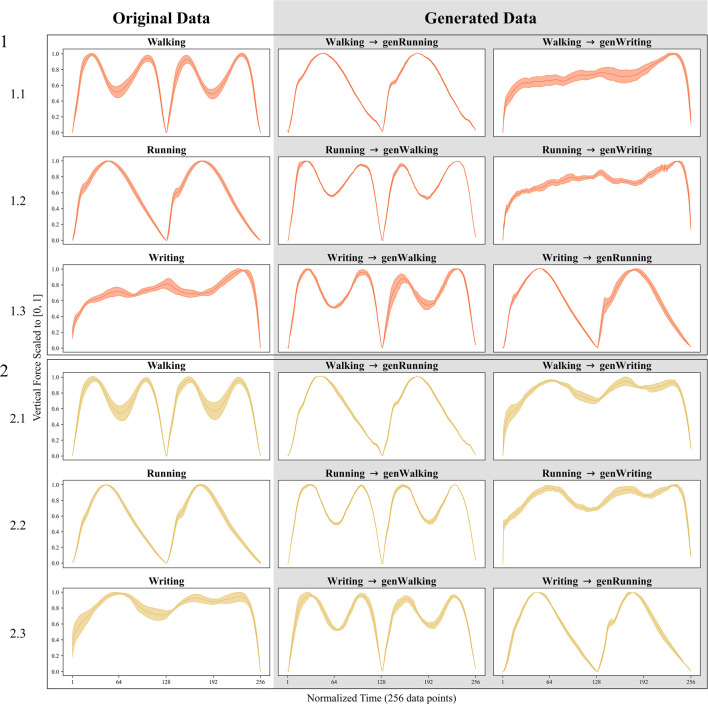
Original and CycleGAN-generated Data. Shown is the test set data of Participant 3 (1) and Participant 5 (2). Panels 1.1 and 2.1 show the original walking data and the genRunning and genHandwriting data generated from them. Accordingly, panels 1.2 and 2.2 refer to the original running data, and panels 1.3 and 2.3 to the original handwriting data each with the data generated from them. The mean values and standard deviation over the respective curves are shown in each case. On the *x*–axis, the respective courses over the 256 time points are shown. The *y*–axis shows the vertical force scaled to the interval [0, 1]. For the walking and running data, the values 1 to 128 represent the ground contact of the left foot and the values 129 to 256 of the right foot. genRunning, generated running data; genWalking, generated walking data; and genWriting, generated handwriting data.

Figure 4 shows an example of the data of the participants p3 (Figure 4.1) and p5 (Figure 4.2). Looking at the shape of the curves, it is noticeable that the generated running (genRunning), generated walking (genWalking), and generated handwriting (genWriting) data correspond to the curve of the respective original data. However, based on the examples, it is noticeable that genWalking and genRunning data generated from walking or running data show a distribution with lower variance. In addition, the examples of genRunning data from walking data show a small wave at the end of the left and right ground contact, which does not occur in the original running data. In the genWalking and genRunning data, which are based on the handwritten data, it can also be seen that there are sections within the curves that vary noticeably in variance (e.g., Figure 4.1.3: the right ground contact in each case for genWalking and genRunning). A more detailed look at the genWriting data shows that they deviate somewhat more from the original data than genRunning data and genWalking data. In addition, for example, there are also fewer smooth curve components (Figure 4.1.2). Furthermore, it is noticeable that the first value of the generated data tends to be too small (i.e., by 0.0) and does not match the original data (i.e., by 0.2).

Looking at the data at the individual participant level, we see that individual characteristics of the original walking, running, and handwriting data were carried over into the genWalking, genRunning, and genWriting data, respectively. For example, in both p3 and p5, the somewhat stronger impact peak of the right ground contact during running is also transferred accordingly in the respective genRunning data. In addition, in the genWalking data at p3 (Figure 4.1.2), the left ground contact shows a higher loading peak than the terminal stance peak, which is exactly the opposite in the right ground contact but corresponds to the original data in both cases. Also, in handwriting, for example, it can be seen that the pattern of p5 (Figure 4.2.3) is somewhat wavier than that of p3 (Figure 4.1.3), which is also reflected in the corresponding genWriting data.

### 3.3 Classification with generated test data

Table 4 presents the results of the person classifications with generated test data. The classification F_1_–score of the genRunnings generated from walking data and the genWalkings from running data was 92.5% and 98.9%, respectively. The classification F_1_–score of the genRunnings and genWalkings from handwriting data was 78.7% and 78.4%, respectively. For the genWriting data generated from the walking and running data, the F_1_–score was 46.8% and 50.0%, respectively. Thus, the results are 7.7–14.0 times better than the ZRB guess probability.

**TABLE 4:** Classification results of person recognition using SVM with generated test data.

Classification problem		Classification score [%]					Number of trials	
Test Data	Generated from	Acc	F1	Prec	Rec	ZRB	Train	Test
genRunning	←Walking	93.6	92.5	95.9	93.6	6.7	1,636	1,067
genWriting	←Walking	52.2	46.8	49.7	52.2	6.0	101	1,067
genWalking	←Running	98.9	98.9	98.9	98.9	7.5	1,067	1,636
genWriting	←Running	53.9	50.0	50.1	53.9	6.0	101	1,636
genRunning	←Writing	71.3	78.7	75.5	86.7	6.7	1,636	101
genWalking	←Writing	73.3	78.4	73.4	88.1	7.5	1,067	101

ZRB, Zero–Rule Baseline; Acc, Accuracy; F_1_, F_1_–score; Prec, Precision; Rec, Recall; genWalking, Generated walking data; genRunning, Generated running data; and genWriting, Generated handwriting data.

## 4 Discussion

### 4.1 Person classification based on original data

To test the underlying assumption of the individuality of the collected walking, running, and handwriting patterns in the present study, person classifications were performed. The results of the person classifications based on the original data confirm our assumption that both vertical GRF in walking or running and vertical pen pressure in handwriting clearly differ between participants in each case (F_1_–score: running: 99.6%, walking: 99.7%, and writing: 99.0%). These results are therefore within the range of previous studies on the individuality of movement patterns (Bauer and Schöllhorn, 1997; Schöllhorn et al., 2002a; Schöllhorn et al., 2002b; Schöllhorn et al., 2006; Janssen et al., 2008; 2011; Schmidt, 2012; Albrecht et al., 2014; Horst et al., 2016; Horst et al., 2017a; Horst et al., 2017b; Horst et al., 2019; Burdack et al., 2020a).

In order to be able to relate the results of the person classifications between the movements based on the original data, the data sets of the walking and running data were adjusted to the size of the data set of the writing data. Due to the reduction of the data set, the classification results of the original walking (99.7%–98.0% F_1_–score) and running (99.6%–99.0% F_1_–score) data slightly decreased. When comparing the classification results between the vertical GRF of walking and running and the vertical pen pressure patterns in handwriting, no differences were shown between the signals of running and those of writing. However, there is a difference, albeit very slight, between the results based on the signals of walking and those of walking or writing. A possible explanation for the somewhat more individual patterns in running compared to walking could be due to, for example, a different frequency spectrum or higher applied forces (Burdack et al., 2020a). Similar to the study by Schöllhorn et al. (2002a), where the most extreme heel heights of shoes resulted in the highest recognition rate, running can be seen as a more extreme movement that forces the participants to show their individuality. Whereas in walking speed there are many more possibilities for compensation. However, for larger data sets, the above points seem to play a minor role in influencing person classification. One approach to explaining the slightly better handwriting results compared to those of walking could be the different localization of the movement control in the central nervous system. The time normalization and accompanying possible interpolation of the vectors should have only a subordinate influence on the results of the classification (Burdack et al., 2020b).

### 4.2 Data generation and person classification based on generated data

In the first step of data analysis, transformations between the vertical GRF data of walking and running movements and the vertical pen pressure of handwriting were learned using the deep learning method CycleGAN (Zhu et al., 2017) with a participant’s class conditioning. Then, based on the learned transformations, the data of each of the other two movements was artificially generated from the third movement. This generated data was then tested in a person classification trained with the original data.

The results ranged from 46.8% to 98.9% F1–score, corresponding to almost 8 times and up to 14 times the guess probability, respectively. Consequently, this provided the first evidence that it is possible to learn pairwise transformations between the respective movement data on the one hand, and preservation of individual structures on the other hand.

The generation of the genRunning (92.5% F_1_–score) and genWalking (98.9% F_1_–score) data from the original walking and running data worked particularly well. This impression can be confirmed by looking at the figures (Figure 4) of the generated data. The generated genWalking and genRunning data from the original walking and running data are not only very similar in their general shape to the original data but also reflect the respective individual characteristics such as impact peak or time course features in their curves. However, the figures (Figure 4) also show differences between generated and original data. For example, the variance of the generated data was shown to be significantly lower than that of the original data. This observation could be attributed to the fact that GANs aim to learn probability distributions that accurately represent the underlying data generation process (Goodfellow et al., 2014; Goodfellow et al., 2020). Thus, the generator might have tried to stay as close as possible to the learned probability distribution. In addition, curve features were also uncovered that did not appear to have a substantial effect on classification but did not match the original data. On the one hand, this provides a reason for caution for the use of the generated data, and on the other hand, it reveals optimization potential that should be addressed in future research.

To be able to explain the approximated 6% difference in the classification results of the genRunning and genWalking data, several explanatory approaches come into question. One possible approach could be due to the highly non–convex optimization process of the conditional CycleGAN so that the generator was stuck in a local minimum, for example, or the initially learned weights negatively influenced the learning process of the genRunnings. Alternatively, it could be that the running data contains more or different information than the walking data, and it is therefore easier for the conditional CycleGAN to generate genWalking data from the running data than genRunning data from the walking data. However, if we put the classifications in relation to the guessing probability, we notice that the results with 13.2 and 13.8 times the ZRB roughly correspond.

The results of the genRunning and genWalking data from handwriting data are 78.7% and 78.4% F_1_–score and the genWriting data from running (50.0% F_1_–score) and walking (46.1% F_1_–score) data are at least 7.7 times better than the ZRB. Thus, the results are worse than those of the genRunning and genWriting data generated from the walking and running data. The figures (Figure 4) provide a first explanation in this respect. At first glance, the genRunning and genWalking data from the handwriting data and the genWriting data from the running and walking data correspond quite closely to the original data, including the adoption of the curve–specific individual characteristics. The genRunning and genWalking data from the handwriting data, for example, show a relatively strong unequal distribution of the variance in certain curve segments, which does not occur in the original data. However, this could also be due to the relatively small amount of handwriting data generated so that individual trials with a greater deviation from the mean are more significant and are shown relatively overrepresented. The genWriting data also shows a lower variance than the original handwriting data and represents the original curve in part only in a rough form. A further potential explanation for the lower classification results compared to the data generated between walking and running, as well as the discrepancy between the genRunning and genWalking data and the genWriting data derived from walking and running data, could be attributed to the quantity of handwriting data. The limited training data available for the conditional CycleGAN could result in the generator learning a probability distribution that does not accurately represent the original data. One more possible explanation is that generating handwriting is inherently more challenging than generating walking or running. Additionally, the bipedal nature of walking and running may provide additional information about the relationship between the left and right steps, which could facilitate the generation, but this is not present in the case of handwriting. To what extent the different central nerves control locomotion movements such as walking or running and arm or wrist movements such as writing influences the identification of individual patterns remain a subject of future research. The extent to which analyzing the first letter of the sentence while handwriting, and a possible altered variation in movement associated with this, may also have influenced the results remains a subject for future investigation. A further possible explanation could lie in the architecture and training parameters of the conditional CycleGAN so that parameter tuning or optimization of the architecture of the generator or discriminator (details in chapter IV.D) can achieve domain–specific improvements.

### 4.3 Identification of underlying individuality across movements

The results provide further evidence for the possibility of automatic recognition of movement patterns across movements. So far, this could only be done for very similar movements using the joint angle curves in shot put, discus, and javelin throwing, taking into account all kinematic variables except that of the throwing arm (Horst et al., 2020). With the proposed method, we can extend this approach considerably. As shown in this work, transferable individual movement features can also be found in movement data that differ significantly in their time course and originate from very different movements.

Other previous studies on the automatic identification of individual movement patterns investigated these in each case only based on single movement signals (Bauer and Schöllhorn, 1997; Schöllhorn et al., 2002a; Schöllhorn et al., 2002b; Schöllhorn et al., 2006; Janssen et al., 2008; Janssen et al., 2011; Schmidt, 2012; Albrecht et al., 2014; Horst et al., 2016; Horst et al., 2017a; Horst et al., 2017b; Horst et al., 2019; Burdack et al., 2020a).

Even though we can provide the first cross–movement approaches in this work, we are only at the beginning of cross–movement research. We were able to provide the first evidence in this study, using vertical forces and pressure data respectively, that it is possible to learn transformations between these data to generate artificial data that still preserve latent patterns of the original data. Thus, we provide a “proof of concept” of the presented method, which has the potential to represent a starting point for further research in this context.

In this respect, an extension of the study to other data signals as well as other biological data would be useful. It would be of interest to see how additional movement components such as three-dimensional GRF, which has been shown to be better than vertical GRF in classifying individuals (Horst et al., 2023), could convey additional information about the individual. While a transfer from kinematic data to GRF data during walking could be shown using GANs (Bicer et al., 2022), there is still great potential to be exploited cross–movement wise. For example, it is important to find out the extent to which the large thematic overlap of locomotion movements might affect inherent biases related to movement character or motor and neurological control compared to handwriting. It is therefore even more encouraging that more different movement signals, such as that of handwriting, could be adequately generated. This gives room for an experimental extension to other signals as well as to linkages with other signals such as audio (e.g., voice), ECG, or EEG (John et al., 2022), which, however, remain the subject of future research.

Furthermore, while we have found individual cross–movement commonalities, what these explicitly look like and what characteristics they exhibit should be addressed in future research. In addition to the individual movement component, it might also be possible to find further latent patterns or subcomponents across movements. Other movement components could be the movement technique or situational adaptations to fatigue, emotions, environment, etc.

The individual patterns across movements also suggest that there might be characteristic features of a person that are at least reflected in several movements. Whether a link can be established between movement and the psychological characteristics of a person could also be the subject of future work. In addition, it remains to be investigated whether individual movement patterns behave similarly and are consistent with patterns found in behavioral research (Buss and Plomin, 1975; Funder and Colvin, 1991; Sherman et al., 2010; Marcus and Roy, 2017). This could be of specific interest for economizing training or therapy. Whether changing the gait by training or therapy, which is sometimes observed after psychotherapy, and whether this has an effect on the handwriting or *vice versa* could be one area of a more holistic approach to future practical applications.

### 4.4 Data generation and cross–movement analysis by conditional CycleGANs

The methodology presented in this paper has overcome the problem of mapping two biomechanical time series signals to each other while transferring the individual component. It was thus possible to learn the transfer of movement A to movement B while obtaining latent patterns of movement A. This could provide fundamental new opportunities in future experiments where one is looking for latent structures between movements or movement–signals. In the following, we discuss optimization potentials and application possibilities of conditional CycleGANs.

First, it must be emphasized restrictively that in the context of this study, the person condition of the CycleGAN was necessary to learn the transformation including the preservation of the individual component from movement A to movement B. Specifically, this means that it was not possible, for example, to generate a person’s individual handwriting data from the walking data without prior knowledge of that person’s handwriting.

For the conditional CycleGANs, the amount of walking (approximately 10,000 steps), running (approximately 14,000 steps), and handwriting data (approximately 900 trials) appears to be sufficient to learn transformations between movements based on vertical ground reaction forces or vertical pen pressure. However, the results suggest that the quality of the learned transformation depends largely on the amount of data such that possibly the 900 handwritten trials were too few to produce deceptively real trials, while the amount of walking and running data provided a good basis to perform person classification with very high recognition rates; the generated curves also showed “errors”. In order to use data for generation, domain–specific optimizations would need to be made (Saxena and Cao, 2022) so that the generated data are not only indistinguishable using a classifier but make biological sense and are indistinguishable from original data by experts. Apart from a larger data set, we see the greatest optimization potential in adjusting the learning rate of the generator’s optimizer (especially in relation to the learning rate of the discriminator). In addition, the number of epochs, or the selected batch size also seems to provide the potential for domain–specific optimization.

The particular potential of the conditional CycleGAN lies in the possibility of cross–movement analysis due to the learnability of transformations from one movement signal to another movement signal. In addition, as in the setting of the present work, the conditional CycleGAN could be used to identify further latent patterns across movements. In doing so, it would also be possible to search for cross–person abstract patterns of, for example, fatigue, emotion, or illness. Another potential of the conditional CycleGAN could be especially in an area where it is difficult to collect large amounts of data. There, artificial augmentation of small data sets could open up the possibility of using data–intensive methods (e.g., deep learning approaches and machine–learning classification). The advantage of the conditional CycleGAN in this context is that this algorithm requires relatively little data and that the training data need not be paired, or the construction of artificial pairs is obsolete.

## 5 Conclusion

In recent years, the analysis of movement patterns has increasingly focused on the individuality of movements, revealing individual patterns with situation–dependent fine structures. However, previous research methods only allowed the comparison of very similar movement signals. In this study, we were able to identify similarities between individual walking, running, and handwriting patterns across different movements through data augmentation, revealing individual patterns across movements. This further extends the understanding of strong individuality.

Based on the results of movement science studies that use machine learning methods to investigate the uniqueness of individual movement patterns, and the findings presented in this study, it can be inferred that our understanding of the individuality of human movement and the influence of individuality on targeted development, improvement, or recovery is still in its beginning stages. Understanding individual cross–movement commonalities in movement may offer insights into the underlying more general individuality of the central nervous system physiology and structure. Future applications of this approach have the potential to investigate the extent to which the central nervous system or muscle physiology can be altered beyond the individual domain.

In addition, this study provides proof of concept that it is possible to use the conditional CycleGAN to artificially generate cross–movement data with latent movement characteristics of the original movement without relying on paired data. In summary, the methodology presented in this study helps to enable cross–movement analysis and artificially generate larger data sets.

## Data Availability

The raw data supporting the conclusion of this article will be made available by the authors, without undue reservation.
