# Supplementary material for: Identifying underlying individuality across running, walking, and handwriting patterns with conditional cycle–consistent generative adversarial networks
Source: Front Bioeng Biotechnol. 2023 Aug 4;11:1204115. doi: 10.3389/fbioe.2023.1204115 (PMC10436554; doi:10.3389/fbioe.2023.1204115)
Supplement: Supplementary file 1 [file DataSheet1.docx]

Supplementary Material

Identifying Underlying Individuality Across Running, Walking, and Handwriting Patterns with Conditional Cycle–Consistent Generative Adversarial Networks

Johannes Burdack^*^, Sven Giesselbach, Marvin L. Simak, Mamadou L. Ndiaye, Christian Marquardt, Wolfgang I. Schöllhorn

*** Correspondence:** Johannes Burdack, burdack@uni-mainz.de

# Supplementary Data

## Supplementary Tables

Table S1. Description of the Data Sizes in the Whole Analysis Process Depending on the Different Classification Tasks.

| Classification Problem | | Cond. CycleGAN | | SVM | |
| --- | --- | --- | --- | --- | --- |
| Generated Test Data | Generated from Original Data | Number of Training Data | | Number of Trials | |
|  |  |  |  | Train (Original Data) | Test  (Generated Data) |
| genWalking | Running | Walking | 9,594 | 1,067  (62.9 ± 12.3) | 1,636  (96.3 ± 18.1) |
|  |  | Running | 14,722 |  |  |
| genRunning | Walking | Running | 14,722 | 1,636  (96.3 ± 18.1) | 1,067  (62.9 ± 12.3) |
|  |  | Walking | 9,594 |  |  |
| genWalking | Writing | Walking | 9,594 | 1,067  (62.9 ± 12.3) | 101  (5.9 ± 0.25) |
|  |  | Writing | 905 |  |  |
| genWriting | Walking | Writing | 905 | 101  (5.9 ± 0.25) | 1067  (62.9 ± 12.3) |
|  |  | Walking | 9594 |  |  |
| genRunning | Writing | Running | 14,722 | 1,636  (96.3 ± 18.1) | 101  (5.9 ± 0.25) |
|  |  | Writing | 905 |  |  |
| genWriting | Running | Writing | 905 | 101  (5.9 ± 0.25) | 1,636  (96.3 ± 18.1) |
|  |  | Running | 14,722 |  |  |

A SVM with 10–fold Cross–Validation for original walking and running data and 5–fold for original writing data was applied. The table shows the total number of trials for the training and test data from all 17 participants. The mean amount and standard deviations of the trials of each participant are shown in round brackets. genRunning = generated running data, genWalking = generated walking data, genWriting = generated handwriting data.

Table S2. Calculation of the Zero Rule Baseline for each Classification Problem.

| Classification  Problem | Number of Training Trials | | ZRB |
| --- | --- | --- | --- |
|  | Total | Most Frequent Class |  |
| Walking | 9,594  [905] | 720  [54] | 720/9,594 = 7.5%  [54/905 = 6.0%] |
| Running | 14,722  [905] | 986  [54] | 986/14,722 = 6.7%  [54/905 = 6.0%] |
| Writing | 905 | 54 | 54/905 = 6.0% |
| genRunning from Walking | 1,636 | 110 | 110/1,636 = 6.7% |
| genWriting from Walking | 101 | 6 | 6/101 = 6.0% |
| genWalking from Running | 1,067 | 80 | 80/1,067 = 7.5% |
| genWriting from Running | 101 | 6 | 6/101 = 6.0% |
| genRunning from Writing | 1,636 | 110 | 110/1,636 = 6.7% |
| genWalking from Writing | 1,067 | 80 | 80/1,067 = 7.5% |

Presented is the calculated Zero Rule Baseline (ZRB) score depending on the most frequent class of the respective training set for each classification task. ZRB = Number of Training Trials of the most frequent class / total training trials. As a comparison the baseline classification in balanced classes would be 1 divided by the number of classes (in our case: 1/17 = 5.9%). genRunning = generated running data, genWalking = generated walking data, genWriting = generated handwriting data.
